# Hypovitaminosis D Does Not Aggravate the Progression of Gentamicin-Induced Kidney Injury in Rats

**DOI:** 10.3390/diseases13070200

**Published:** 2025-06-28

**Authors:** Ana Lívia D. Maciel, Amanda L. Deluque, Beatriz M. Oliveira, Cláudia S. Souza, Heloísa D. C. Francescato, Cleonice Giovanini, Francisco J. A. de Paula, Terezila M. Coimbra, Rildo A. Volpini

**Affiliations:** 1 Laboratory of Renal Physiology, Department of Physiology, Ribeirao Preto Medical School, University of Sao Paulo, Ribeirao Preto 14040-900, SP, Brazil; diasanalivia@usp.br (A.L.D.M.); alimadel@ualberta.ca (A.L.D.); drabeatrizmagalhaes@gmail.com (B.M.O.); cau.souzaa07@gmail.com (C.S.S.); helodcf@gmail.com (H.D.C.F.); cleonis2003@yahoo.com.br (C.G.); tmcoimbr@fmrp.usp.br (T.M.C.); 2 Department of Internal Medicine, Ribeirao Preto Medical School, University of Sao Paulo, Ribeirao Preto 14049-900, SP, Brazil; fjpaula@fmrp.usp.br

**Keywords:** gentamicin, kidney injury, hypovitaminosis D

## Abstract

**Background/Objectives**: Gentamicin is one of the most effective and widely used antibiotics to treat serious infections. In addition to its bactericidal properties, gentamicin has a nephrotoxic effect that results in acute kidney injury (AKI). AKI may be intensified by hypovitaminosis D. This study evaluated the effect of hypovitaminosis D in the progression of gentamicin-induced renal injury. **Methods**: Male Wistar Hannover rats received a standard (SD) or a vitamin D-free diet (VitD^—^) before gentamicin treatment. After that, we divided the animals into four groups: Ctrl VitD, SD diet, and saline injection; Ctrl VitD^—^, VitD^—^ diet, and saline injection; Genta VitD, SD diet, and gentamicin injection (40 mg/kg; IM); Genta VitD^—^, VitD^—^ diet, and gentamicin injection (40 mg/kg; IM). After the end of gentamicin treatment, we followed the animals for 5 days (protocol 1) and 30 days (protocol 2). **Results**: The Genta VitD group (protocol 1) presented impaired renal function. Regarding morphological analyses, the Genta VitD group presented necrotic tubules (protocol 1) and atrophied tubules (protocol 2). In the inflammatory scenario, the Genta VitD group presented an increase in the number of CD68+ cells, as well as in the levels of interleukin 1β (protocols 1 and 2). In addition, gentamicin-treated animals (protocols 1 and 2) presented an increased renal expression of vimentin and fibronectin. Despite the notable changes in functional, inflammatory, and structural parameters induced by gentamicin, hypovitaminosis D did not aggravate the renal injury in this experimental model. **Conclusion**: Hypovitaminosis D did not aggravate the progression of gentamicin-induced renal injury in rats.

## 1. Introduction

Gentamicin is an aminoglycoside antibiotic commonly used against serious infections caused by gram-negative bacteria [1]. In addition to its bactericidal properties, gentamicin is nephrotoxic, affecting 10 to 25% of patients who use this medication [2]. The exact mechanism of nephrotoxicity is not well understood, but tubular cytotoxicity is one of the central aspects [3]. In the cytoplasm, gentamicin alters cell function by acting directly and indirectly on mitochondria, interrupting the respiratory chain and impairing ATP production, activating the intrinsic apoptosis pathway and increasing oxidative stress [4].

Gentamicin also modifies tubular reabsorption by inhibiting several cell membrane transporters in the brush border and basolateral membrane [5], including Na^+^/Pi cotransporter [6], Na^+^/H exchanger [7], and Na^+^/K^+^/ATPase [8]. Furthermore, there is evidence that the pathogenesis of gentamicin-induced renal damage is related to inflammation and generation of reactive oxygen species (ROS) [9]. In addition, evidence in experimental model has shown that after recovering from gentamicin-induced acute kidney injury (AKI), there is an accumulation of renal interstitial collagen resulting in renal fibrosis, with increased expression of transforming growth factor beta 1 (TGF-β1), endothelin, angiotensin II, alpha smooth muscle actin (α-SMA), and macrophages in the renal cortex [10,11]. The process that leads to renal fibrosis occurs when the tissue suffers an injury, which triggers the generation of a pro-fibrotic field that favors the recruitment, proliferation, and activation of myofibroblasts [12].

Hypovitaminosis D (deficiency and insufficiency) is recognized as a relevant public health problem, with approximately 1 billion people worldwide being affected by altered vitamin D status [13]. This paradox appears to be the result of several factors that influence the biological production of vitamin D, such as inadequate sun exposure or use of protective factors, environmental factors (high latitude regions, prolonged winter), physiological factors (dark skin pigmentation, advanced age, pregnancy, liver and kidney diseases), and inadequate intake/supplementation of this hormone [14]. Among these populations at greater risk of presenting hypovitaminosis D, older patients, obstetric women, and neonates comprise the populations most susceptible to the use of gentamicin [15].

In recent decades, it has been demonstrated that vitamin D is a factor that can directly interact with AKI, since its reduced levels can accelerate the development of the injury, while AKI can dysregulate vitamin D homeostasis and its respective function [16]. Thus, the dysregulation of vitamin D may act as a predisposing factor, a concomitant event, or a consequence of kidney dysfunction. During episodes of AKI, there is a significant loss of kidney function, which affects the renal enzymatic activity responsible for vitamin D activation, potentially resulting in a deficiency of this hormone [17]. On the other hand, vitamin D depletion may promote the activation of the renin–angiotensin–aldosterone system (RAAS), leading to increased production of angiotensin II. This activation results in elevated blood pressure, damage to the renal microvasculature, and worsening of kidney function [18].

Furthermore, studies in different models of kidney disease have shown that vitamin D has potent anti-inflammatory effects, highlighting that the administration of vitamin D can reduce the infiltration of inflammatory cells and prevent the formation of interstitial fibrosis [19,20,21]. On the other hand, several studies using different experimental models of kidney injury have shown that vitamin D insufficiency is one of the factors that can intensify and/or contribute to the progression of kidney disease [22,23,24]. Based on that, we investigated the influence of hypovitaminosis D in a murine model of gentamicin-induced renal injury.

## 2. Materials and Methods

### 2.1. Animals

Male Wistar Hannover rats, weighing 180–200 g, were provided by the animal facility from the University of Sao Paulo—Ribeirao Preto Campus. We kept our animals at a controlled temperature (22 ± 1 °C) with a light/dark cycle of 12/12 h. All the experiments followed our institutional guidelines and were approved by the local Research Ethics Committee (CEUA, registration 106/2021).

### 2.2. Diets

We used two different types of diet in our experimental protocols: (1) Standard diet (SD) protocol AIN93G (including 1000.0 IU/kg Vitamin D_3_), and (2) Vitamin D-free diet (VitD^—^), both manufactured by Pragsoluções Biociências (Jaú, Brazil). The only variable in the composition between the two diets is the presence or absence of vitamin D.

### 2.3. Experimental Design

In previous studies conducted by our research group, we found typical changes of the acute phase of nephrotoxicity at 5 days after gentamicin-treatment. After 30 days, despite the renal function recovering, we noted persistent areas of morphological changes in renal tissue, which related to the formation of renal fibrosis [11,25]. These findings demonstrate that the experimental model used reflects the progression of acute kidney injury induced by gentamicin [11,25]. In the present protocol, the animals underwent a one-week adaptation period. After that, we divided the animals into two groups, each one submitted to a specific diet (SD or VitD^—^) for six consecutive weeks. After this period, we used 40 mg/kg/day of gentamicin sulfate (Schering-Plough S/A, Brazil) during six consecutive days, intramuscularly (IM) to perform our experimental model of nephrotoxic acute kidney injury. The control group received saline injection (NaCl 0.9%) under the same conditions as gentamicin. After that, we divided the animals into four groups: control + standard diet (Ctrl VitD, n = 7); control + vitamin D free diet (Ctrl VitD^—^, n = 7); gentamicin + standard diet (Genta VitD, n = 9); and gentamicin + vitamin D free diet (Genta VitD^—^, n = 9). At the end of these treatments, we monitored the animals for another 5 days (protocol 1) or 30 days (protocol 2), maintaining the respective diets until the end of each protocol.

In the next step, we weighed and anesthetized the animals with ketamine (0.05 mL/100 g) and xylazine (0.1 mL/100 g) via intraperitoneal (IP) injection (Cristália, Itapira, Brazil). We cannulated the abdominal aorta for blood collection (stored at −70 °C) and removed the kidney. Fragments of renal tissue were fixed in Methacarn’s solution (60% methanol, 30% chloroform and 10% acetic acid) for 24 h. After this period, the fixative was replaced with 70% alcohol and we used the renal tissue for histological and immunohistochemical studies. The remaining renal tissue was stored at −70 °C for Western blot and enzyme-linked immunosorbent assay (ELISA) studies.

### 2.4. Renal Function Studies

Before euthanasia, we placed the rats from both protocols in individual metabolic cages, on a 12/12 h light/dark cycle, with free access to drinking water and diets. We collected 24 h urine to assess urinary output and then centrifuged the samples to remove suspended material. We evaluated urine osmolality by freezing point depression method (Fiske Osmometer, Norwood, MA, USA), creatinine (colorimetric method using picric acid), and sodium by the ion-selective electrode quantification technique (Electrolyte Analyzer, Roche Diagnostics GmbH, Mannheim, Germany).

### 2.5. 25 Hydroxyvitamin D [25(OH)D_3_], Calcium, and Phosphorus Levels

Quantitative determination of 25(OH)D_3_ was performed on serum samples collected from both protocols. We used the direct competitive test based on the chemiluminescence principle (CLIA) (DiaSorin, Liaison^®^, Saluggia, Italy). We also measured plasma levels of calcium and phosphorus by the colorimetric method (Kit Labtest Diagnóstica S.A, Lagoa Santa, Brazil).

### 2.6. Histological Studies

Renal tissue was embedded in paraffin and sliced into 4 µm-thick histological sections. We stained the microscope slides with Masson’s trichrome and examined under light microscopy (AxioVision Rel. 4.3; Zeiss, Oberkochen, Germany). We analyzed 30 consecutive fields (0.1 mm^2^) in the renal cortex, and determined the number of tubules with necrosis and atrophied tubule as well. We considered as acute tubular necrosis (ATN) features: necrosis of tubular cells, dilatation of the tubular lumen due to flattening of proximal tubular cells with loss of the brush border, and vacuolization of tubular cells [26].

### 2.7. Immunohistochemical Analysis

Renal tissue sections were deparaffinized and hydrated. After that, we incubated the sections for 20 min with normal goat serum to block nonspecific antigen binding. Then, we incubated the renal tissues with the following primary antibodies: anti-vimentin (1/500, MO 725, Dako Corporation, Glostrup, Denmark) for 60 min at room temperature; anti-ED1 (CD68) (1/1000, MCA341R, Serotec, Kidlington, UK); and anti-fibronectin (1/500, Chemicon International Inc., Temecula, CA, USA) incubated overnight at 4 °C. The reaction product was detected with the avidin-biotin-peroxidase complex (Vector Laboratories, Burlingame, CA, USA) and staining was triggered by the addition of DAB (3,3-diaminobenzidine) (Sigma Chemical Company, St. Louis, MO, USA) in the presence of hydrogen peroxide. Counterstaining was with methyl green, and the sections were then dehydrated and mounted.

We evaluated 30 consecutive fields of 0.1 mm^2^ in the renal cortex to analyze the immunoreaction for vimentin, fibronectin, and ED1. The expression of vimentin and fibronectin was performed using a scoring method based on the extent of the stained area: score 0, 0–5% of the renal cortex area; score 1, 5–25%; score 2, 25–50%; score 3, between 50 and 75%; and score 4, more than 75% of the analyzed field with immunoreaction [26]. For ED1, we counted the number of CD68+ cells per field in the renal cortex. To minimize bias, the observer was blinded to the treatment groups.

### 2.8. Total Protein Isolation

Kidney samples were homogenized using a lysis buffer 50 mM Tris-HCl, pH 7.4; 150 mM NaCl; 1% Triton X-100; 0.1% SDS (sodium dodecyl sulfate); 1 µg/mL aprotinin; 1 µg/mL leupeptin; 1 mM phenylmethylsulfonyl fluoride; 1 mM sodium orthovanadate, pH 10; 1 mM sodium pyrophosphate; 25 mM sodium fluoride and EDTA (0.001 M ethylenediaminetetraacetic acid, pH 8). Homogenates were centrifuged at 10,000 rpm for 20 min at 4 °C to remove nuclei and cell debris. The supernatant was separated for protein determination and quantified by the Bradford method [27]. Renal tissue lysates were used for Western blot and ELISA experiments.

### 2.9. Western Blot Assay

For Western blot analysis, 60 µg of total renal proteins were separated on a 12% polyacrylamide gel, transferred to a nitrocellulose membrane, and blocked for 60 min with 5% skim milk diluted in TBST (Tris, NaCl, EDTA/L, and Tween-20). Blots were then incubated overnight at 4 °C with the primary antibodies anti-VDR (VDR sc-13133, 1:500, Santa Cruz Biotechnology, Dallas, TX, USA) and anti-CYP24A1 (H00001591-M02, 1:500; Abnova, Walnut, CA, USA). As a reference protein, we utilized GAPDH (Glyceraldehyde-3-phosphate dehydrogenase), using the anti-GAPDH antibody (2118L, monoclonal 1:1000, Sigma Chemical Company, St. Louis, MO, USA). The membranes were washed and incubated with anti-mouse monoclonal IgG antibodies (1:5000, Dako, Denmark) conjugated to peroxidase for 1 h at room temperature. Finally, the imaging system Kodak Gel Logic 2200 (Kodak, Rochester, NY, USA) detected the reaction result of the membrane-bound antibodies after enhanced chemiluminescence (ECL) reaction (Sigma-Aldrich, St. Louis, MO, USA). The intensity of each band was quantified by densitometry and analyzed by the NIH Image J software version 1.53k (National Institute of Health, Research Services Branch, Bethesda, MD, USA). The results are expressed as a percentage of the ratio between the protein of interest and the reference protein in relation to the control group (Ctrl VitD), which was considered 100%.

### 2.10. Evaluation of IL-1β

Using the renal tissue lysates, we quantified the tissue levels of interleukin-1 beta (IL-1β) by the ELISA method using a commercial kit (R&D Systems Inc, Minneapolis, MN, USA). We determined the protein levels in the renal tissue by the Bradford method [27] and the results were expressed in pg/mg of protein.

### 2.11. Statistical Analysis

The data set for each analysis was subjected to the Kolmogorov–Smirnov distribution normality test. For variables with normal distribution, we applied analysis of variance and the Newman–Keuls multiple comparison tests. Data without normal distribution were analyzed by the nonparametric Kruskal–Wallis test followed by Dunn’s post hoc test. We performed statistical analyses using Graph Pad Prism version 9.0 Windows software (Graph Pad Software, La Jolla, CA, USA). Data were expressed as mean ± standard error of the mean (SEM). The level of statistical significance considered was *p* < 0.05.

## 3. Results

### 3.1. Body Weight

Treatment with gentamicin did not alter the body weight of animals in protocol 1. However, the animals from the Genta VitD and Genta VitD^—^ groups (protocol 2) presented a significant reduction in body weight compared to the Ctrl VitD group (Table 1).

### 3.2. Renal Function

Protocol 1 (5 days): The Genta VitD and Genta VitD^—^ groups presented significant increased plasma creatinine levels, fractional excretion of sodium, urinary flow, and significant decreased glomerular filtration rate compared to the control groups (Ctrl VitD and Ctrl VitD^—^) (Table 1). In addition, the Genta VitD^—^ group presented a significant decrease regarding urine osmolality (U_osm_) compared to the Ctrl VitD and Ctrl VitD^—^ groups. Furthermore, the Genta VitD^—^ group presented a significant increase in plasma creatinine compared to the Genta VitD group (Table 1).

Protocol 2 (30 days): The Genta VitD group presented significant higher plasma creatinine levels compared to the Ctrl VitD (Table 1). Regarding the other functional parameters, we did not find any changes in the studied groups (Table 1).

### 3.3. Serum Vitamin D Levels and Plasma Calcium and Phosphorus Levels

The Ctrl VitD^—^ and Genta VitD^—^ groups presented significantly lower levels of 25(OH)D_3_ compared to the Ctrl VitD and Genta VitD groups in both protocols (Table 2). In protocol 1, we found lower plasma calcium levels in the Genta Vit D^—^ group compared to the Genta Vit D group (Table 2). Regarding protocol 2, we did not notice any differences in plasma calcium and phosphorus levels (Table 2).

### 3.4. Western Blot Assay—VDR and CYP24A1

**Figure 1:**
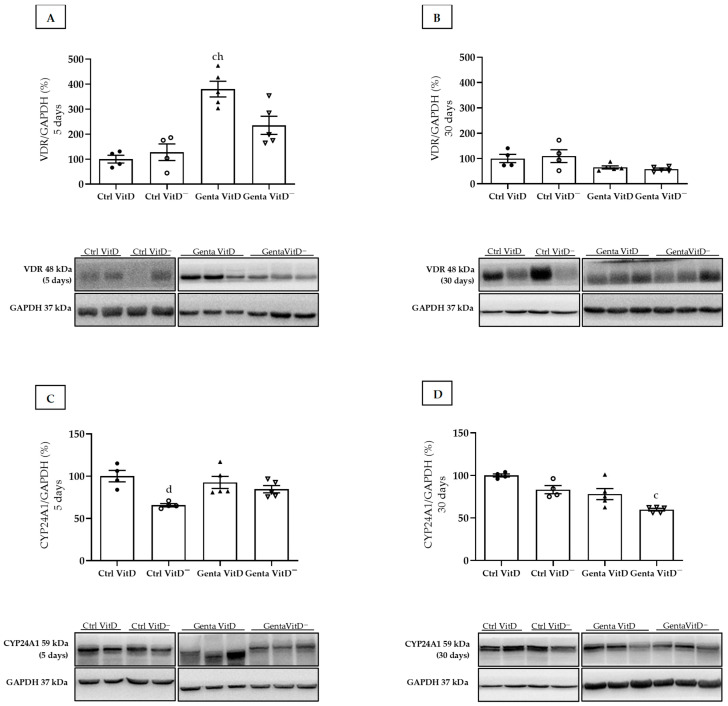
Expression of vitamin D receptor (VDR) and CYP24A1 assessed by Western blot at end of protocols 1 (5 days) and 2 (30 days) of rats fed a standard diet (VitD) or a VitD-free diet (VitD^—^) and treated with saline (Ctrl) or gentamicin (Genta). Densitometric analysis and representative bands of immunoblots that reacted with anti-VDR in protocol 1 (**A**) and protocol 2 (**B**) and for CYP24A1 in protocol 1 (**C**) and protocol 2 (**D**). Densitometric ratio was calculated among bands corresponding to VDR and CYP24A1 in relation to GAPDH, with data expressed in relation to Ctrl VitD, which was considered 100%. Data are expressed as mean ± SEM. ^c^ *p* < 0.01, ^d^ *p* < 0.05 vs Ctrl VitD; ^h^ *p* < 0.05 vs Ctrl VitD^—^.

The Genta VitD group (protocol 1) presented an increase in renal VDR expression compared to the Ctrl VitD and Ctrl VitD^—^ groups (Figure 1A). In protocol 2, we did not find any differences in VDR expression (Figure 1B). In addition, we used CYP24A1 as another marker of the total renal vitamin D content. In protocol 1, the Ctrl VitD^—^ group presented a lower CYP24A1 expression compared to the Ctrl VitD (Figure 1C). In protocol 2, we noted a lesser CYP24A1 enzyme expression in the Genta VitD^—^ group compared to the Ctrl VitD group (Figure 1D).

### 3.5. Histological Analysis

**Figure 2:**
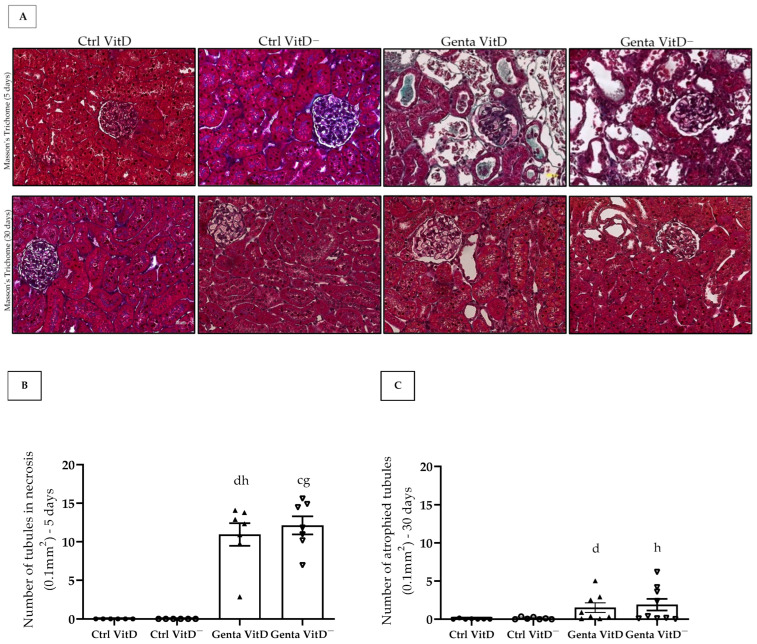
(**A**) Histological sections stained with Masson’s Trichome of rats fed a standard diet (VitD) or a VitD-free diet (VitD^—^) treated with saline solution (Ctrl) or gentamicin (Genta) from protocols 1 (5 days) and 2 (30 days). 400× magnification. Number of necrotic tubules of animals from protocol 1 (**B**) and number of atrophied tubules of animals from protocol 2 (**C**). Data are expressed as mean ± SEM. ^c^ *p* < 0.01, ^d^ *p* < 0.05 vs. Ctrl VitD; ^g^ *p* < 0.01, ^h^ *p* < 0.05 vs. Ctrl VitD^—^.

We found the presence of tubular necrosis in both Genta VitD and Genta VitD^—^groups from the protocol 1 (Figure 2A), which showed features of ATN, characterized by sloughing of cells in the tubular lumen, denudation of the basement membrane, and loss of the brush border [26]. In addition, we noted a significant difference among gentamicin-treated groups and saline-treated groups (Figure 2B). In protocol 2, the Genta VitD and Genta VitD^—^ groups presented an increased number of atrophied tubules (Figure 2A,C). Also, the gentamicin-treated groups presented a statistical difference from the saline-treated groups (Figure 2A,C).

### 3.6. Immunohistochemistry Analysis

#### 3.6.1. Vimentin

**Figure 3:**
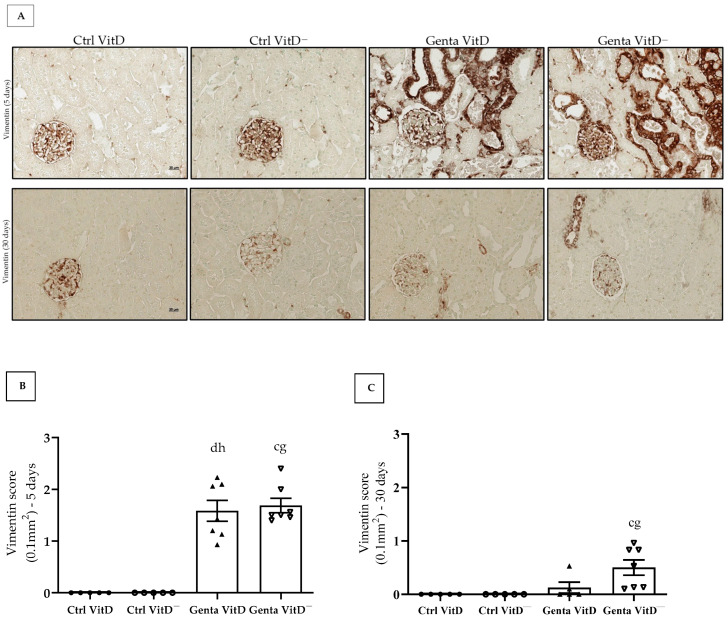
(**A**) Immunolocalization of vimentin in renal cortex of rats from protocols 1 (5 days) and 2 (30 days) fed a standard diet (VitD) or a VitD-free diet (VitD^—^) treated with saline (Ctrl) or gentamicin (Genta). Magnification 400×. Vimentin score obtained from protocol 1 (**B**) and protocol 2 (**C**). Data are expressed as mean ± SEM. ^c^ *p* < 0.01, ^d^ *p* < 0.05 vs. Ctrl VitD; ^g^ *p* < 0.01, ^h^ *p* < 0.05 vs. Ctrl VitD^—^.

In protocol 1, the Genta VitD and Genta VitD^—^ groups presented a significant increase in vimentin expression compared to the Ctrl VitD and Ctrl VitD^—^ groups (Figure 3A,B). In protocol 2, we only noticed a more intense vimentin expression in the renal cortex of the Genta VitD^—^ group compared to the Ctrl VitD group (Figure 3A,C).

#### 3.6.2. Fibronectin

**Figure 4:**
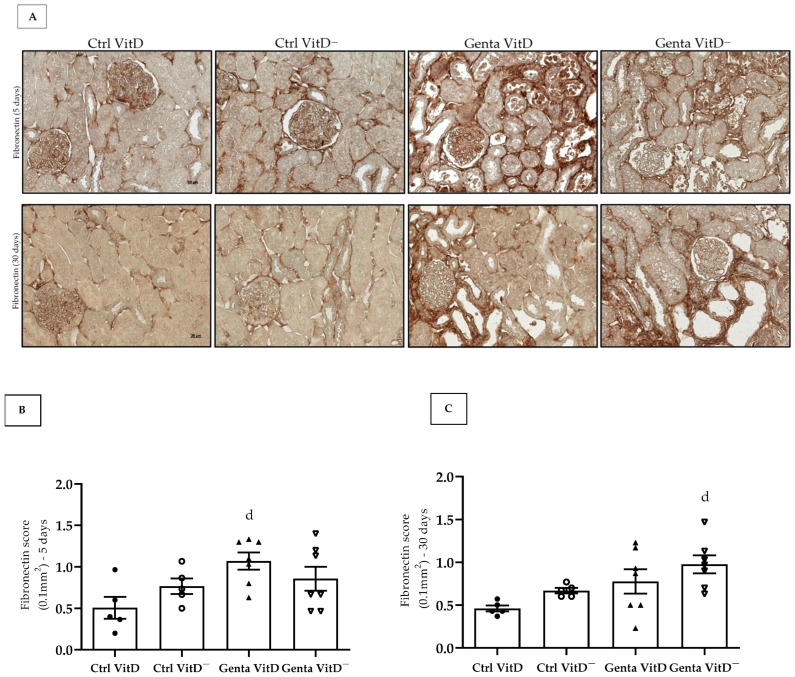
(**A**) Immunolocalization for fibronectin in renal cortex of rats from protocols 1 (5 days) and 2 (30 days) fed with standard diet (VitD) or VitD-free diet (VitD^—^) treated with saline (Ctrl) or gentamicin (Genta). Magnification 400×. Fibronectin score obtained from protocol 1 (**B**) and protocol 2 (**C**). Data are expressed as mean ± SEM. ^d^ *p* < 0.05 vs. Ctrl VitD.

Fibronectin is an extracellular matrix protein and its increased expression is generally related to the process of tissue fibrosis. In protocol 1, we found an increased fibronectin expression in the renal tissue in the Genta VitD group compared to the Ctrl VitD group (Figure 4A,B). In protocol 2, we noted that the Genta VitD^—^ group presented a significant more intense labeling for fibronectin compared to the Ctrl VitD group (Figure 4A,C).

#### 3.6.3. ED1—CD68+ Cells (Macrophages)

**Figure 5:**
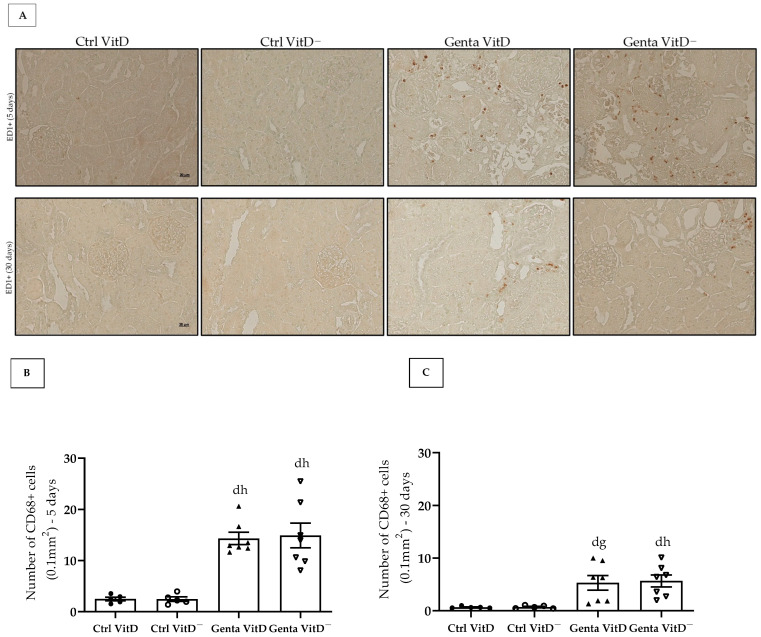
(**A**) Immunolocalization of CD68+ cells in renal cortex of rats from protocols 1 (5 days) and 2 (30 days) fed with standard diet (VitD) or VitD-free diet (VitD^—^) treated with saline (Ctrl) or gentamicin (Genta). Magnification 400×. Quantification of the number of CD68+ cells obtained from protocol 1 (**B**) and protocol 2 (**C**). Data are expressed as mean ± SEM. ^d^ *p* < 0.05 vs. Ctrl VitD; ^g^ *p* < 0.01, ^h^ *p* < 0.05 vs. Ctrl VitD^—^.

In protocol 1, the Genta VitD and Genta VitD^—^ groups presented a significant increase in the number of CD68+ cells compared to the Ctrl VitD and Ctrl VitD^—^ groups (Figure 5A,B). Although to a lesser extent, the animals from the Genta VitD and Genta VitD^—^ groups (protocol 2) presented a higher number of CD68+ cells compared to the Ctrl VitD and Ctrl VitD^—^ groups (Figure 5A,C).

### 3.7. Quantification of Interleukin 1β (IL-1β)

**Figure 6:**
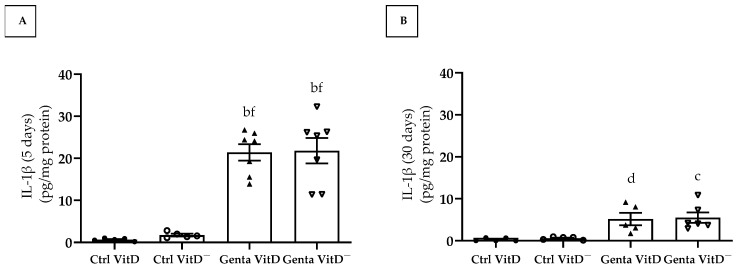
Quantification of IL-1β levels (ELISA) in renal cortex of rats from protocols 1 (5 days) (**A**) and 2 (30 days) (**B**) fed a standard diet (VitD) or a VitD-free diet (VitD^—^) treated with saline (Ctrl) or gentamicin (Genta). Data are expressed as mean ± SEM. ^b^ *p* < 0.001, ^c^ *p* < 0.01, ^d^ *p* < 0.05 vs. Ctrl VitD; ^f^ *p* < 0.001 vs. Ctrl VitD^—^.

In protocol 1, the animals from the Genta VitD and Genta VitD^—^ groups presented a significant increase regarding IL-1β levels compared to the Ctrl VitD and Ctrl VitD^—^ groups (Figure 6A). In protocol 2, we found significant higher IL-1β levels in the Genta VitD and Genta VitD^—^ groups in comparison to the Ctrl VitD and Ctrl VitD^—^ groups (Figure 6B).

## 4. Discussion

In our experimental model of nephrotoxicity, the Genta VitD group (protocol 1) presented increased fractional excretion of sodium and urinary flow, in addition to a decreased glomerular filtration rate. We found no statistical differences regarding these parameters in relation to the Genta VitD^—^ group (protocol 1). Concerning the morphological analysis, the Genta VitD group presented alterations in the renal structure, such as the presence of necrotic tubules (protocol 1) and atrophied tubules (protocol 2). In the inflammatory scenario, the Genta VitD group displayed an increased number of CD68+ cells as well as in the interleukin 1β levels (protocols 1 and 2). We found no significant differences in renal morphology and inflammatory parameters compared to the Genta VitD^—^ group (protocols 1 and 2). Our data provide evidence that hypovitaminosis D did not aggravate gentamicin-induced renal injury in rats.

In general, plasma vitamin D levels represent the sum of production resulting from skin exposure to ultraviolet B (UVB) radiation, dietary intake, and foods fortified with vitamin D [28]. Our groups of animals were kept in a controlled environment and received a diet depleted or not in vitamin D. The groups fed the vitamin D-free diet (Ctrl VitD^—^ and Genta VitD^—^) had insufficient plasma vitamin D levels compared to the Ctrl VitD and Genta VitD groups of both protocols 1 and 2, confirming the efficiency of our experimental model of hypovitaminosis D [24,29,30].

Another marker used to indicate renal vitamin D content is the expression of 24-hydroxylase (CYP24A1), since its function is to prevent the accumulation of high levels of vitamin D by degrading its metabolites [31]. Our results for CYP24A1 demonstrated that the renal expression of this enzyme was significantly lower in the Ctrl VitD^—^ group (protocol 1) and in the Genta VitD^—^ (protocol 2). CYP24A1 is highly induced by calcitriol, and in conditions of low vitamin D levels, there is a reduction in the expression of this enzyme and a consequent decrease in the degradation of vitamin D. This fact reflects a compensatory mechanism for an increase in serum levels of this hormone in the body [32].

The exact mechanism of gentamicin nephrotoxicity is not well understood, but tubular cytotoxicity is one of the central aspects [3]. The aminoglycoside-megalin/cubilin complex undergoes endocytosis [33] and gentamicin accumulates in the intracellular space, triggering several processes that alter cell function and result in tubular, glomerular, and vascular damage [34,35,36]. In the cytoplasm, gentamicin alters cell function by acting directly and indirectly on the mitochondria, interrupting the respiratory chain and impairing ATP production, activating the intrinsic apoptosis pathway, and increasing oxidative stress [4].

Gentamicin also modifies tubular reabsorption by inhibiting several cell membrane transporters in the brush border and basolateral membrane, regardless of cell damage [5]. The result of this process is a reduction in the excretory function of the affected nephrons and an increase in hydrostatic pressure inside the tubule and Bowman’s capsule, leading to a reduction in the filtration pressure gradient and a consequent decrease in the glomerular filtration rate (GFR) [35].

Hypovitaminosis D is common in kidney disease [37] and one of its effects is associated with impaired kidney function, as already demonstrated in several experimental [24,30,38] and clinical studies [39,40,41]. Vitamin D has a bidirectional relationship with AKI, since its reduced levels can accelerate the development of the injury, while AKI per se can dysregulate vitamin D homeostasis and its respective function [16].

To assess renal function, we quantified some parameters, such as plasma creatinine, fractional excretion of sodium, glomerular filtration rate, and urine osmolality. Our Genta VitD and Genta VitD^—^ groups presented increases in plasma creatinine levels, fractional excretion of sodium, and urinary flow, as well as a decrease in glomerular filtration rate and urine osmolality. In addition, we noticed that hypovitaminosis D significantly increased plasma creatinine levels in the Genta VitD^—^ group compared to the Genta VitD group (protocol 1). Unlike our finding, de Bragança et al. observed in an acute ischemia/reperfusion (IRI) model that vitamin D-deficient animals did not present elevated plasma creatinine levels and reduced GFR after IRI. These results obtained by the authors show that vitamin D deficiency did not modify the functional parameters evaluated at the end of 2 and 7 days after IRI [22].

Although we had found an increase in plasma creatinine levels in the Genta VitD^—^ group, the respective GFR of this group of animals did not show a significant difference in relation to the Genta VitD group. This result allows us to speculate that, although insufficient, the vitamin D levels still detectable in the Genta VitD^—^ group were probably able to prevent the decline in renal function in this group of animals. Corroborating our findings, Gonçalves et al. did not either observe a decline in the glomerular filtration rate (GFR) evaluated in a model of renal disease progression in vitamin D-deficient animals subjected to ischemia/reperfusion injury [23]. On the other hand, a study by Park et al. on vitamin D supplementation in an experimental protocol involving gentamicin and paricalcitol demonstrated that this vitamin D analogue improved kidney function [42].

The biological activity of vitamin D depends on its binding to its nuclear receptor (VDR), whose response results in the regulation of transcription in target cells [43]. The regulation of VDR expression occurs by genetic and environmental factors [44]. The regulation mechanism is related to the binding of calcitriol to VDR, since this binding protects this receptor from the proteasomal degradation process, resulting in its greater bioavailability. Renal cells are capable of synthesizing vitamin D, but in a situation of renal damage, there is a reduction in calcitriol levels, decreasing the binding of this isoform to VDR. Thus, this receptor becomes more susceptible to degradation, resulting in a decrease in its expression [45,46]. In our study, we noted a significant increase of VDR expression in renal tissue only in the Genta VitD group from the protocol 1. Mohamed et al. found that animals treated with gentamicin and supplemented with vitamin D_3_ presented increased VDR expression in renal tissue. The authors also described that the group of animals supplemented with vitamin D_3_ had an increase in the expression of 8-OHDG (8-hydroxyguanosine), an important biomarker of oxidative stress, which led to the understanding that a decreased expression of VDR can aggravate the oxidative stress process caused by gentamicin. supplementation with vitamin D_3_ had an upregulation on the expression of 8-OHDG (8-hydroxyguanosine), an important biomarker of oxidative stress, which led to the understanding that a decreased VDR expression can aggravate the oxidative stress process caused by gentamicin [47]. In addition, several studies, which investigated the effect of vitamin D deficiency in different experimental models of renal injury, showed a decrease in VDR expression [24,38]. Gonçalves et al. associated decreased VDR amount with increased TGF-β1 expression in the renal tissue of vitamin D-deficient animals subjected to ischemia/reperfusion, relating this result to the renal damage and formation of renal fibrosis [23]. Although these studies have shown that vitamin D deficiency can reduce VDR expression, we did not find this condition in our results in either experimental protocol. It is important to highlight that the aforementioned studies showed that vitamin D levels were undetectable, since the experimental protocols were long-term. In the present study, the detectable levels of vitamin D in the animals of our protocols 1 and 2 were probably sufficient to maintain renal VDR expression, contributing to a lower proteasomal degradation of this receptor.

In addition to functional changes, treatment with gentamicin can promote morphological changes in renal tissue [11,25], which is rarely evaluated in the clinical setting. The evaluation of the renal structure revealed that our groups treated with gentamicin showed morphological changes such as ATN in protocol 1 and the presence of atrophied tubules in protocol 2. Previous experimental studies with gentamicin corroborate our findings, which also observed the presence of necrotic tubules with dilation of the lumen due to flattening of the proximal tubular cells, loss of the brush border, and vacuolization of tubular cells, events that result in the process of ATN [4,48,49]. Albino et al. observed generalized ATN on day 1 after administration of gentamicin and regeneration of tubular cells on days 30 and 180 [10]. Furthermore, Mohammed et al. demonstrated that prior administration of vitamin D, two weeks before treatment with gentamicin, prevented changes in the renal structure in the initial phase of the injury [47]. Although many studies have suggested that hypovitaminosis D impairs the tubular injury repair process [22,23], we only noticed slight changes (not significant) in renal tissue in Genta VitD^—^ groups compared to Genta VitD (protocols 1 and 2). Gonçalves et al. observed no differences in GFR in vitamin D-deficient animals analyzed 62 days after renal ischemia/reperfusion insult. On the other hand, the authors found significant differences in the renal morphology of these animals, showing that vitamin D deficiency can aggravate the progression of renal disease in the long term [23]. Such evidence corroborates our findings regarding the levels of vitamin D still detectable in our experimental model. In other words, the levels of vitamin D still detectable were probably sufficient to mitigate the progression of renal injury.

Gentamicin-induced renal damage involves an inflammatory response at the site of injury, increasing the migration of macrophages to the spot of tissue damage [50,51]. Our results demonstrate that the animals treated with gentamicin from both protocols presented an increase in the number of CD68+ cells (macrophages) and in the levels of the pro-inflammatory cytokine IL-1β in the renal tissue. In the initial phase of the injury (protocol 1), our animals treated with gentamicin presented a significant amount of CD68+ cells in the renal tissue. In protocol 2, the CD68+ cell infiltrate persisted, although to a lesser extent. In addition, we did not find changes regarding renal IL-1β levels between the Genta VitD^—^ and the Genta VitD groups (protocols 1 and 2). Many studies have been demonstrating that vitamin D can modulate the production of pro-inflammatory cytokines, such as tumor necrosis factor-α, interleukin-6, interferon gamma, and IL-1β, while stimulating the synthesis of anti-inflammatory cytokines, such as interleukin-10 [52,53]. Previous studies from our research group demonstrated that calcitriol-treated rats presented a decreased number of CD68+ cells and lower IL-1β levels in the renal cortex in AKI models [21,32], reinforcing the role of vitamin D in modulating the inflammatory process. Although our animals fed the vitamin D-free diet had insufficient levels of vitamin D, these were still detectable and probably sufficient not to intensify the inflammatory process in our model of gentamicin-induced nephrotoxicity.

After an AKI insult by gentamicin that results in functional and structural damage and activation of inflammatory pathways, residual areas of fibrosis may persist in the renal tissue [10,11]. As previously reported, AKI can result in incomplete tissue repair, persistent tubulointerstitial inflammation, fibroblast proliferation, excessive deposition of extracellular matrix (ECM) components, and phenotypic modification [54,55]. Fibrosis is a process that results in the accumulation of ECM proteins such as fibronectin and collagen, whose main mediator of this process is TGF-β [56]. Previous experimental studies with gentamicin have associated increased renal fibrosis with high TGF-β1 levels in the kidney [11,57,58]. In the present study, we did not find significant differences in the renal expression of fibronectin and vimentin (a marker of phenotypic alteration) in the Genta VitD and Genta VitD^—^ groups from either protocol 1 and 2. Several studies have demonstrated that vitamin D status can modulate the expression of ECM components and markers of phenotypic alteration [19,20,21,23,24,29,38]. Although we had noted a mild increase in the renal expression of fibronectin and vimentin in the Genta VitD^—^ group, we considered that the hypovitaminosis D present in our experimental model was not sufficient to aggravate the renal injury induced by gentamicin. These results corroborate our findings regarding the functional data and inflammation markers analyzed in both protocols studied. Therefore, our results as whole emphasize the importance of using animal models as a translational pathway to support clinical practice.

## 5. Conclusions

Taken together, our results demonstrate that experimentally gentamicin-induced nephrotoxicity modifies renal function and structure. Moreover, hypovitaminosis D did not aggravate the progression of renal injury in rats monitored 5 and 30 days after discontinuation of gentamicin treatment.

## Figures and Tables

**Table 1:** Renal function parameters obtained at the end of protocols 1 (5 days) and 2 (30 days) of rats fed with standard diet (VitD) or vitamin D-free diet (VitD^—^) and treated with saline (Ctrl) or gentamicin (Genta).

	Ctrl VitD	Ctrl VitD^—^	Genta VitD	Genta VitD^—^
BW (g)				
5 days 30 days	0.340 ± 0.006 0.364 ± 0.020	0.333 ± 0.011 0.321 ± 0.010	0.310 ± 0.008 0.307 ± 0.012 ^d^	0.308 ± 0.008 0.312 ± 0.009 ^d^
P_creat._ (mg/dL)				
5 days 30 days	0.39 ± 0.01 0.40 ± 0.03	0.51 ± 0.03 0.50 ± 0.03	2.25 ± 0.32 ^c,g^ 0.67 ± 0.02 ^c^	3.64 ± 0.64 ^a,e,k^ 0.66 ± 0.03
TFG (ml/min/100g)				
5 days 30 days	0.39 ± 0.07 0.47 ± 0.07	0.20 ± 0.04 0.45 ± 0.05	0.10 ± 0.03 ^d^ 0.28 ± 0.01	0.10 ± 0.04 ^d^ 0.24 ± 0.06
FE_Na+_ (%)				
5 days 30 days	0.20 ± 0.02 0.20 ± 0.02	0.29 ± 0.03 0.28 ± 0.04	1.01 ± 0.35 ^c^ 0.42 ± 0.02	0.94 ± 0.34 ^d^ 0.54 ± 0.17
U_osm_ (mOsm/KgH_2_O)				
5 days 30 days	1173 ± 66.5 1069 ± 246	1227 ± 206 1007 ± 231	595 ± 63.6 955 ± 149	529 ± 58.5 ^d,h^ 1032 ± 130
V (ml/min)				
5 days 30 days	0.004 ± 0.001 0.006 ± 0.002	0.004 ± 0.0008 0.004 ± 0.001	0.016 ± 0.001 ^a,e^ 0.007 ± 0.001	0.016 ± 0.002 ^a,e^ 0.008 ± 0.001

P_creat_: plasma creatinine; GFR: glomerular filtration rate; FE_Na+_: fractional excretion of sodium; U_osm_: Urine osmolality; V: Urine flow rate. Data are expressed as mean ± SEM. ^a^ *p* < 0.0001, ^c^ *p* < 0.01, ^d^ *p* < 0.05 vs. Ctrl VitD; ^e^ *p* < 0.0001, ^g^ *p* < 0.01, ^h^ *p* < 0.05 vs. Ctrl VitD^—^; ^k^ *p* < 0.05 vs. Genta VitD.

**Table 2:** Serum vitamin D levels and plasma calcium and phosphorus levels obtained at the end of protocols 1 (5 days) and 2 (30 days) of rats fed with standard diet (VitD) or VitD^—^free diet (VitD^—^) and treated with saline solution (Ctrl) or gentamicin (Genta).

	Ctrl VitD	Ctrl VitD^—^	Genta VitD	Genta VitD^—^
25(OH)D_3_ (ng/mL)				
5 days 30 days	42.5 ± 1.37 38.3 ± 1.99	11.00 ± 1.44 ^b^ 8.66 ± 0.77 ^a^	38.7 ± 1.93 ^f^ 39.9 ± 2.57 ^e^	12.5 ± 0.77 ^b,j^ 9.68 ± 0.86 ^a,i^
P_Ca_ (mg/dL)				
5 days 30 days	11.6 ± 0.45 11.90 ± 0.18	10.8 ± 0.43 11.70 ± 0.03	13.50 ± 0.52 ^c,f^ 11.60 ± 0.20	10.6 ± 0.27 ^j^ 11.00 ± 0.35
P_P_ (mg/dL)				
5 days 30 days	5.41 ± 0.19 5.51 ± 0.32	5.91 ± 0.38 5.29 ± 0.42	5.18 ± 0.22 5.60 ± 0.46	5.39 ± 0.31 5.69 ± 0.78

25(OH)D_3_: 25 hydroxyvitamin D_3_; P_Ca_: Plasma calcium concentration; P_P_: Plasma phosphorus concentration. Data are expressed as mean ± SEM. n = 7–9 for each group. ^a^ *p* < 0.0001, ^b^ *p* < 0.001, ^c^ *p* < 0.01 vs. Ctrl VitD; ^e^ *p* < 0.0001, ^f^ *p* < 0.001 vs. Ctrl VitD^—^; ^i^ *p* < 0.0001, ^j^ *p* < 0.001 vs. Genta VitD.

## Data Availability

Data are contained within the article.
